# A new species of *Loxosceles* Heineken & Lowe (Araneae, Sicariidae), with updated distribution records and biogeographical comments for the species from Mexico, including a new record of *Loxoscelesrufescens* (Dufour)

**DOI:** 10.3897/zookeys.802.28445

**Published:** 2018-12-04

**Authors:** Alejandro Valdez-Mondragón, Mayra R. Cortez-Roldán, Alma R. Juárez-Sánchez, Karen P. Solís-Catalán

**Affiliations:** 1 CONACYT Research Fellow. Laboratory of Arachnology (LATLAX), Laboratorio Regional de Biodiversidad y Cultivo de Tejidos Vegetales (LBCTV), Instituto de Biología, Universidad Nacional Autónoma de México (UNAM), sede Tlaxcala, Ex-Fábrica San Manuel, San Miguel Contla, 90640 Santa Cruz Tlaxcala, Tlaxcala, Mexico Universidad Nacional Autónoma de México Tlaxcala Mexico; 2 Colección Nacional de Arácnidos (CNAN), Departamento de Zoología, Instituto de Biología, Universidad Nacional Autónoma de México (UNAM), Ciudad Universitaria, Apartado Postal 04510, Coyoacán, Mexico City, Mexico Universidad Nacional Autónoma de México Tlaxcala Mexico; 3 Laboratory of Arachnology (LATLAX), Laboratorio Regional de Biodiversidad y Cultivo de Tejidos Vegetales (LBCTV), Instituto de Biología, Universidad Nacional Autónoma de México (UNAM), sede Tlaxcala, Ex-Fábrica San Manuel, San Miguel Contla, 90640 Santa Cruz Tlaxcala, Tlaxcala, Mexico Universidad Nacional Autónoma de México Tlaxcala Mexico

**Keywords:** Biogeography, *Loxoscelesmalintzi* sp. n., North America, taxonomy

## Abstract

A new species of the spider genus *Loxosceles* Heineken & Lowe, 1832, *Loxoscelesmalintzi***sp. n.**, is described from the states of Puebla, Morelos and Guerrero, in the central region of Mexico. The description is based on adult males and females with morphological and ultra-morphological images. Updated distribution maps are provided for the 39 species recorded from the Mexican territory (including the new species). The states with the greatest diversity are Baja California Sur, Baja California and Sonora, with five species each. A total of 441 records for the 39 species, based on arachnological collections, data bases and literature, were used to update the distribution maps. *Loxoscelesboneti* Gertsch, 1958 is the species with the highest number of records in Mexico, with a total of 58 records from different localities. The states with the most records so far are Guerrero, with 55 records, Morelos, with 35 records, and Baja California Sur, with 30 records. *Loxoscelesrufescens* (Dufour, 1820), an introduced species, is recorded for the second time in Mexico, from the state of Chihuahua, being the first well-documented record for the country. Mexico has the greatest diversity of species of *Loxosceles* worldwide, with 39 (two introduced species) of the 134 described species. Additionally, biogeographical comments for the species from Mexico are provided.

## Introduction

Spiders of the genus *Loxosceles* Heineken & Lowe, 1832 are better known in North America as “violin spiders”, “recluse spiders”, or “brown recluse spiders”; commonly known by the medical community and general public to cause dermonecrotic lesions caused by their poisonous bites and the venom component, Sphingomyelinase D, an enzyme that destroys endothelial cells lining the blood vessels (Vetter and Barger 2002; Vetter and Bush 2002; Vetter et al. 2003, 2009; Wendell 2003; Da Silva et al. 2004; Vetter 2005, 2008, 2015; Sandidge and Hopwood 2005; Ramos-Rodríguez and Méndez 2008; Manríquez and Silva 2009; Swanson and Vetter 2009). The genus *Loxosceles* belongs to the spider family Sicariidae Keyserling, 1880, which comprises three genera: *Hexophthalma* Karsch, 1879, with six species from Africa, *Sicarius* Walckenaer, 1847, with 21 species distributed in Central and South America, and *Loxosceles*, with 134 described species worldwide (Magalhães et al. 2017; World Spider Catalog 2018). Recently, Souza and Ferreira (2018) described the first troglomorphic species of *Loxosceles* from caves of Brazil. According to Binford et al. (2008), species of *Loxosceles* are classified into eight species groups: *reclusa, laeta, amazonica, gaucho, spadicea, rufescens, vonwredei* and *spinulosa*. However, the species group *amazonica* was merged with the species group *rufescens* by Duncan et al. (2010) based on molecular data. The *reclusa* group has the highest diversity, with 50 species, all from North America, primarily Mexico (Gertsch and Ennik 1983). Mexico has the highest diversity of recluse spiders worldwide, with 39 recorded species, of which 37 are native (including the new species herein described) and two are introduced species: *Loxoscelesreclusa* Gertsch & Mulaik, 1940 and *Loxoscelesrufescens* (Dufour, 1820) (Gertsch 1958, 1973; Gertsch and Ennik 1983; World Spider Catalog 2018). The first species described from Mexico was *Loxoscelesyucatana* Chamberlin & Ivie, 1938 from the Yucatan Peninsula. The most complete systematic revision for North American species of *Loxosceles* was published by Gertsch and Ennik (1983), describing 20 new species from Mexico. Thus, this was the last and most complete taxonomic revision for the species that occur in the country. The most recent taxonomic contribution for the species of *Loxosceles* from Mexico was the description of the male of *Loxoscelesmulege* by Jiménez and Llinas (2005) from Baja California Sur.

Some North American synanthropic species of *Loxosceles*, such as *L.reclusa* in the United States, have been closely studied for their biological, medical and physiological aspects, analyzing their abundances, distribution and natural history (Vetter and Barger 2002; Vetter and Bush 2002; Vetter et al. 2003, 2009; Wendell 2003; Vetter 2005, 2008; Sandidge and Hopwood 2005; Swanson and Vetter 2009). However, these aspects are poorly known for species from Mexico. It is not yet known whether the introduced synanthropic species collected in houses and buildings may also be collected in natural areas around the houses. In 2017, four collectors collected around 40 *Loxoscelesmisteca* in two hours from a house in the state of Tlaxcala, Mexico. However, the species has never been collected in natural areas in the state (Valdez-Mondragón et al. 2018). This has been previously reported by Fischer and Vasconcellos-Neto (2005) with *L.laeta* and *L.intermedia* from South America, where these spiders are almost absent from natural areas immediately surrounding the infested buildings where they were collected. Additional research is required for the species from Mexico that have been reported from urban areas.

The primary aim of this paper is to describe a new species of *Loxosceles* from the central region of Mexico, distributed in the states of Puebla, Morelos and Guerrero. Additionally, we update the distribution records for the Mexican territory providing new records, including that of *L.rufescens*, an introduced species from the Mediterranean Basin and the Middle East (Nentwig et al. 2017; Tahami et al. 2017). Finally, we discuss the biogeography of the species of *Loxosceles* from Mexico based on biotic provinces.

## Material and methods

The specimens were hand collected and deposited in ethanol (80%) in the Colección Nacional de Arácnidos (CNAN), Institute of Biology, Universidad Nacional Autónoma de México (IBUNAM), Mexico City, and the Laboratorio de Aracnología (LATLAX), Laboratorio Regional de Biodiversidad y Cultivo de Tejidos Vegetales (LBCTV), IBUNAM, Tlaxcala City. The descriptions and observations of the specimens were made using a Zeiss Discovery V8 stereoscope. A Zeiss Axiocam 506 color camera attached to a Zeiss AXIO Zoom V16 stereoscope was used to photograph the specimens. The male palps and female genitalia were dissected in ethanol (80%). The female genitalia were cleaned in potassium hydroxide (KOH-10%) for 5 to 10 minutes. The habitus, female genitalia and palps were submerged in 96% alcohol gel (ethanol) and covered with a thin layer of liquid ethanol (80%) to minimize diffraction during photography (Valdez-Mondragón and Francke 2015). For the electron micrographs, the morphological structures were dissected and cleaned with an ultrasonic cleaner at 20–40 kHz, critical-point dried, and examined at low vacuum in a Hitachi S-2460N scanning electron microscope (SEM). The descriptions were done following Gertsch and Ennik (1983) and Tahami et al. (2017). Morphological nomenclature follows Ramírez (2014), Planas and Rivera (2015) and Magalhães et al. (2017a, b). All measurements are in millimeters (mm). Measurements on electron micrographs are in micrometers (μm). To update the distribution maps, we used literature, databases and networks, mainly of CNAN, LATLAX and the Global Biodiversity Information Facility (GBIF) (http://www.gbif.org). The records of GBIF belong to specimens that where identified by A. Valdez-Mondragón (first author) in 2007 and by W. J. Gertsch (various dates). The specimens were deposited in the CNAN, in Mexico the second representative and diverse biological collection of *Loxosceles* after LATLAX, which was revised. Nine fieldtrips were made to different states in Mexico to collect additional material of different species: Puebla (March and June, 2017), Tlaxcala (April 2017, 2018; May 2018), Hidalgo (May, 2017), Oaxaca (June, 2017), Guerrero (September, 2017), and Oaxaca (March, 2018). The distribution maps were made using QGIS v. 2.18. For georeferencing and corroboration of localities, two programs were used: GeoLocate online version (http://www.museum.tulane.edu/geolocate/) and Google Earth v.7.1.5.1557. The geographic coordinates were transformed from NAD83 to WGS84 on the online site of INEGI: Transformation of coordinates TRANINV (http://www.inegi.org.mx). Geographical coordinates are given in degrees. Photographs, electron micrographs and maps were edited using Adobe Photoshop CS6.

*Abbreviations*:

AME anterior median eyes;

PLE posterior lateral eyes;

PME posterior median eyes.

## Taxonomy

### Family Sicariidae Keyserling, 1880

#### Genus *Loxosceles* Heineken & Lowe, 1832

##### Loxosceles malintzi sp. n.

Taxon classificationAnimaliaAraneaeSicariidae

http://zoobank.org/27AB2D03-1166-4734-9107-47241F5156C2

Figs 1–5 , 6–10 , 18–21 , 22–27 , 28–31 , 32–37 , 38–43 , 44–49 , 50–54 , 55–62 , 63–67

###### Type material.

MEXICO: *Puebla*: male holotype (CNAN-T01262) from 1.5 km NE of Panteón de San Pablo Anicano (18.1355, −90.1010, 1223 m), Municipality San Pablo Anicano, 5.VII.2017, A. Valdez, A. Juárez, M. Cortez, J. Valerdi Cols. (night collecting). Paratypes: 2 males (CNAN-T01263), 2 females (CNAN-T01264, CNAN-T01265), same data as holotype.

###### Other material examined.

MEXICO: *Puebla*: 1 male, 1 female (LATLAX-Ara 0149), 5 males, 6 females, 19 immatures (LATLAX-Ara0148), same data as holotype. 2 males, 1 female, 13 immatures (LATLAX-Ara0125) [10-III-2017, A. Valdez, E. Briones, A, Juárez, M. Cortez, J. Valerdi Cols.], same locality as holotype. 4 females, 2 immatures (LATLAX-Ara 0122) from 3 km S of San Juan Rabozo (18.54062, −98.44353; 1298 m), Municipality Izúcar de Matamoros, 10-III-2017, A. Valdez, E. Briones, M. Cortez, J. Valerdi, M. Sánchez Cols. 24 immatures (LATLAX-Ara0144) [05-VII-2017, A. Valdez, M. Cortez, A. Juárez, J. Valerdi Cols.], same locality. 8 immatures (LATLAX-Ara 0123), from road to Tepenene (18.49335, −98.39623; 1300 m) Municipality Izúcar de Matamoros, 10-III-2017, A. Valdez, E. Briones, M. Cortez, J. Valerdi, M. Sánchez Cols. 1 female, 7 immatures (LATLAX-Ara0146) [05-VII-2017, A. Valdez, M. Cortez, A. Juárez, J. Valerdi Cols.], same locality. 1 male, 3 female, 31 immatures (LATLAX-Ara0145), 1 male (LATLAX-Ara0194) from 2 km S of Agua Escondida (18.54999, −98.45229; 1275 m), Municipality Izucár de Matamoros 05-VII-2017, A. Valdez, M. Cortez, A. Juárez, J. Valerdi Cols.1 male, 1 female, 13 immatures (LATLAX-Ara0124) from Santa Cruz Tejalpa (18.35028, −98.37773; 986 m), Municipality Tehuitzingo, 10-III-2017, A. Valdez, E. Briones, M. Cortez, J. Valerdi, M. Sánchez Cols. 7 immatures (LATLAX-Ara0126) from 9 km NE of Garzones Santa Gertrudis (18.31016, −98.02065; 1686 m), Municipality Acatlán de Osorio, 11-III-2017, A. Valdez, E. Briones, M. Cortez, J. Valerdi, M. Sánchez Cols. 1 male, 6 immatures (LATLAX-Ara0127), 1 female (LATLAX-Ara0185) from 4 km NE of Totoltepec Guerrero (18.26285, −97.84125; 1427 m), Municipality Totoltepec, 11-III-2017, A. Valdez, E. Briones, M. Cortez, J. Valerdi, M. Sánchez Cols. 10 immatures (LATLAX-Ara0147) from 1.5 km SE of Guadalupe Alchipin (18.25741, −98.21145; 1256 m), Municipality Ahuehuetitla, 05-VII-2017, A. Valdez, E. Briones, M. Cortez, J. Valerdi, M. Sánchez Cols. 2 male, 4 female, 28 immatures (LATLAX-Ara0150) from 2 km al S de Petlatzingo (18.05715, −97.9122; 1439 m) Municipality Petlatzingo, 06-VII-2017, A. Valdez, E. Briones, M. Cortez, J. Valerdi, M. Sánchez Cols. *Guerrero*: 1 male, 8 females, 14 immatures (LATLAX-Ara0163) from road to Mexcaltepec viejo (18.42838, −99.54851; 1142 m), Municipality Taxco de Alarcón, 20-IX-2017, A. Valdez, I. Navarro, P. Solís, J. Valerdi Cols. 1 male (CNAN-Ar009171) 2 km W of Ahuelican “Cerro de la Coronilla” (18.01628, −99.52875; 855 m), Municipality Tepecoacuilco de Trujano, 09-I-2009, O. Francke, A. Valdez, C. Quijano, T. López Cols. *Morelos*: 1 male (CNAN-Ar009174), 1 male (CNAN-Ar009176) from Ticumán (18.76111, −99.11917; 960 m), Municipality Tlaltizapán, 24-IX-2011, G. Montiel Col. 1 male (CNAN-Ar009000) from Biological Station El Limón Cuachichinola (18.52641, −98.93343; 1293 m), Municipality Tepalcingo, 21-IX-2012, G. Montiel, D. Barrales, J. Arreguin Cols. 1 male, 2 immatures (CNAN-Ar009001) from Biological Station El Limón Cuachichinola (18.55132, −98.94288; 1434 m), Municipality Tepalcingo, 22-IX-2012, G. Montiel, D. Barrales, J. Arreguin Cols.

###### Etymology.

The species epithet is a noun in apposition and refers to the volcano “La Malinche, *Malintzi* or Matlalcueye” (meaning “blue skirt” in Nahuatl language), a seismically active volcano (4,420 m) of the Transmexican Volcanic Belt, located in the states of Tlaxcala and Puebla. This last state is where the type locality is located.

###### Diagnosis.

*Loxoscelesmalintzi* sp. n. resembles *L.huasteca* Gertsch & Ennik, 1983 and *L.coyote* Gertsch & Ennik, 1983 in having a long, slender male palpal tibia and by the embolus (Gertsch and Ennik 1983: figs 173‒176, 200‒203). However, *L.malintzi* has a more slender palpal tibia (Figs 22–24, 38, 55–62), 4.4× longer than wide (in *L.huasteca* the tibiae is 2.7× longer than wide (Gertsch and Ennik 1983: fig. 200), and *L.coyote* is 2.9× longer than wide (Gertsch and Ennik 1983: fig. 173)). The palpal tibia of *L.malintzi* is nearly straight (Figs 22–24, 55–62) (in *L.huasteca* and *L.coyote* it is more curved ventrally (Gertsch and Ennik 1983: figs 200, 173 respectively)). In dorsal view, the palpal tibia in *L.malintzi* is nearly straight (Fig. 23) (in *L.huasteca* and *L.coyote* it is more curved each side (Gertsch and Ennik 1983: figs 201, 174 respectively)). In retrolateral view, the embolus of *L.malintzi* is straight as in *L.huasteca* (Gertsch and Ennik 1983: fig. 200), but slightly shorter (Figs 24, 25); also, *L.huasteca* has a small spur near tip of embolus (Gertsch and Ennik 1983: fig. 202), which is absent in *L.malintzi* (Figs 25–27, 40, 41). The embolus in *L.coyote* (Gertsch & Ennik, 1983: figs 173, 176) is markedly longer and wider than in *L.malintzi*, which is smaller and more slender (Figs 22, 24–26, 38–41, 55–62). Females resemble *L.colima* Gertsch, 1958 (Gertsch and Ennik 1983: figs 291‒292) and *L.devia* Gertsch & Mulaik, 1940 (Gertsch and Ennik 1983: figs 42‒46) in having long and curved seminal receptacles; however, *L.malintzi* has seminal receptacles finger-shaped, shorter than those of *L.colima* and less curved than those of *L.devia* (Figs 30, 63–67); also, the base of the receptacles in the new species point obliquely and they are closer to each other (Figs 30, 63–67), whereas in *L.colima* and *L.devia*, the base of the receptacles are widely separated (Gertsch and Ennik 1983: figs 42‒46, 291–292).

###### Description.

**Male (holotype) (CNAN-T01262)**: *Measurements*: Total length 9.30. Carapace 4.40 long, 3.90 wide. Clypeus length 0.62. Diameter of AME 0.22, PME 0.24, PLE 0.22; AME-PME 0.26 Labium: length 0.96, width 0.80. Sternum: length 2.30, width 2.10. Leg lengths: I (total 28.20): femur 7.50 / patella 1.60 / tibia 8.90 / metatarsus 8.40 / tarsus 1.80; II (31.45): 8.40 / 1.60 / 9.80 / 9.75 / 1.90; III (24.10): 7.00 / 1.60 / 6.60 / 7.50 / 1.40; IV (26.90): 7.50 / 1.60 / 7.30 / 8.80 / 1.70. Leg formula: 2-1-4-3.

*Prosoma*: Carapace pale orange, longer than wide, pyriform, with small, numerous setae, with well-defined dark brown “violin” pattern dorsally (Figs 5–7, 10, 18, 29), which is reddish brown in the ocular region and markedly dark brown in posterior part (Figs 18, 29). Carapace with three irregular brown spots on each side. Fovea with a dark brown triangular pattern projected towards posterior part (Figs 18, 29). Six eyes in three groups, clypeus brown (Figs 4, 5, 29). Sternum pale orange, longer than wide (Figs 19, 46). Labium reddish, longer than wide, fused to the sternum, rounded in the middle (Fig. 19). Endites pale orange basally, reddish orange distally and white apically. Endites longer than wide, rounded basally, with sparse long setae, becoming shorter distally (Fig. 19).

*Legs*: Coxae pale yellow, gray towards pro- and retrolateral parts (Fig. 19). Legs with scales (seta) (Fig. 32). Trochanters orange. Femora pale orange, paler on femora III and IV (Figs 18, 19, 36). Patellae reddish basally, pale gray distally. Patellae with two ventral lyriform organs (Figs 35, 37). Claws with seven teeth (Figs 33, 34).

*Chelicerae*: Fused basally, chelated chelicerae laminae, reddish orange, stridulatory lines laterally (Figs 44, 45, 49). Fangs reddish orange, with long and thin setae around them (Figs 44, 45, 47, 48). VO on posterior part of the fang (Figs 47, 48).

*Opisthosoma*: Pale orange, darker posteriorly (Figs 18, 19), oval, longer than wide and high (Figs 18, 19). Region of gonopore pale orange, with small setae. Colulus long, pale orange, conical (Fig. 50). Spinnerets pale orange, anterior lateral spinnerets cylindrical and the longest, posterior median spinnerets smallest, with long setae; posterior median spinerets cylindrical and with many long setae (Figs 50, 51). Tracheae opening near posterior margin of opisthosoma (Fig. 53).

*Palps*: Trochanters pale orange, femora brown, long and thin, patellae brown, tibiae reddish orange and almost cylindrical, wider distally than ventrally (Figs 22–24, 38). Tarsus oval, reddish orange, bulb spherical, with long and straight embolus (Figs 22–27, 38–41). Spermatic outlet at the tip of embolus (Figs 42, 43). Embolus with oval cuticular marks (unknown function) (arrows, Figs 42, 43).

**Female (Paratype) (CNAN-T01264)**: *Measurements*: Total length 9.60. Carapace 4.30 long, 3.60 wide. Clypeus length 0.56. Diameter of AME 0.20, PME 0.23, PLE 0.21; AME-PME 0.25 Labium: length 0.87, width 0.67. Sternum: length 2.12, width 1.90. Leg lengths: I (total 19.65): femur 5.35 / patella 1.40 / tibia 5.90 / metatarsus 5.50 / tarsus 1.50; II (19.00): 5.70 / 1.50 / 6.20 / 4.40 / 1.20; III (18.10): 5.20 / 1.40 / 4.70 / 5.40 / 1.40; IV (20.90): 5.90 / 1.40 / 5.60 / 6.50 / 1.50. Leg formula: 4-1-2-3.

Differs from male as follows: *Prosoma*: Carapace pale orange, with well-defined dark brown “violin” pattern (Figs 20, 28). Carapace without three irregular brown spots on each side but with a wide and well-defined dark brown marginal region, forming a pale “bat-wing”-shaped region in the middle (Fig. 28). Sternum darker orange (Fig. 21). Labium more reddish orange, less rounded in the middle. Endites more reddish orange, less rounded basally.

*Legs*: Coxae yellow, paler gray towards pro- and retrolateral parts (Fig. 21). Trochanters darker orange. Femora pale brown (Figs 20, 21). Patellae reddish brown basally, darker gray distally. Tibiae brown, metatarsi and tarsi dark orange (Figs 20, 21).

*Chelicerae*: Darker reddish brown, with stridulatory lines laterally.

*Opisthosoma*: Opisthosoma dark gray (Figs 20, 21). Spinnerets darker orange. [Note: Vetter (2015) mentioned that the opisthosoma color depends what the spider eats, so the coloration is variable].

*Palps*: Trochanters pale orange, femora pale brown, patellae brown, tibiae and tarsi reddish with several long and wide spread setae around. Tibiae cylindrical, tarsi conical (Fig. 20).

*Genital area*: Seminal receptacles visible by transparency in ventral view (Fig. 31). Seminal receptacles asymmetric, finger-shaped (Fig. 30). Right lobe long and curved, with one small accessory lobe receptacle next to it. Left lobe long, less curved than right one, without accessory receptacles. Base of seminal receptacles wide and strongly sclerotized, directed toward each other in oblique position (Fig. 30). See variation section for more details.

###### Variation.

MALES. *Puebla*: Males from San Pablo Anicano are light brown, with brown spots on carapace well marked, legs darker than the body. *Morelos*: Males from Biological Station “El Limón” are light brown, with dark irregular brown spots on carapace, legs same color as carapace. Male from Tlaltizapan is light brown, with light brown spots on carapace, legs darker than the body. *Guerrero*: Male from road to Mexcaltepec Viejo, is light brown, with dark brown spots strongly marked on carapace, pale brown legs. Male from Tepecoacuilco de Trujano, is light brown, with dark brown spots on carapace slightly marked, legs light brown. *Puebla*: Agua Escondida, Municipality of Izúcar de Matamoros (N= 2): Tibia I 7.3, 7.5; carapace length (CL) 3.6, 4.4, carapace width (CW) 3.0, 3.2. 1.5 km NE of Panteón de San Pablo Anicano (*N* = 3): Tibia I 5.9–8.9 (*x* = 8.0), CL 4.1–4.5 (*x* = 4.0), CW 3.6–4.0 (*x* = 4.0). San Pablo Anicano (*N* = 2): Tibia I 7.5–9.0 (*x* = 8.0), CL 4.0–4.4 (*x* = 4.0), CW 3.36–3.7 (*x* = 4.0). *Morelos*: Biological Station “El Limon” (*N* = 2) Tibia I 7.5, 11.0, CL 4.0, 4.2, CW 3.2, 3.9. *Guerrero*: road to Mexcaltepec viejo (*N* = 1): Tibia I 9.0, CL 3.8, CW 3.3; Tepecoacuilco de Trujano (*N* = 1): Tibia I 7.0, CL 3.6, CW 3.2. FEMALES. *Puebla*: females from San Pablo Anicano are light brown on carapace and legs, with a dark brown marginal region on carapace strongly marked. Females from San Juan Rabozo are dark brown, with dark brown marginal region on carapace strongly marked, legs light brown. *Guerrero*: females from road to Mexcaltepec Viejo are brown dark on carapace, with dark brown marginal region on carapace strongly marked, legs the same color as the body. *Puebla*: 1.5 km NE of Panteón de San Pablo Anicano (*N* = 1): Tibia I 6.0, CL 4.2, CW 3.7. San Pablo Anicano (*N* = 3): Tibia I 4.2–5.5 (*x* = 4.6), CL 3.7–4.1 (*x* = 3.9), CW 2.8–3.6 (*x* = 3.2). San Juan Rabozo, Municipality of Izúcar de Matamoros (*N* = 4): Tibia I 4.9–6.1 (*x* = 5.5), CL 3.5–4.3 (*x* = 3.9), CW 3.0–3.7 (*x* = 3.4). *Guerrero*: road to Mexcaltepec viejo (*N* = 4): Tibia I 5.7–6.2 (*x* = 6), CL 3.4–4.4 (*x* = 3.8), CW 3.4–3.7 (*x* = 3.6).

There is little variation in the shape of the male palps, even those from different populations (Figs 55–62). The seminal receptacles of females are asymmetrical and are broadly variable in shape, even in the specimens from the same locality (Figs 63–66). Some specimens have long and wide curved receptacles, finger-shaped (Figs 63, 65), with small accessory lobes receptacles on each side, more visible in some specimens than others (Figs 63, 65). Others have long and thin seminal receptacles (Figs 64, 66). The base of the seminal receptacles is variable; in some specimens wider, rounded and strongly sclerotized, directed towards each other in oblique position, but in other specimens, the base is slightly sclerotized and thinner (Figs 63–67).

###### Remarks.

Gertsch (1958) and Gertsch and Ennik (1983) reported *Loxosceleszapoteca* Gertsch, 1958 (female specimen) and *Loxoscelesboneti* Gertsch, 1958 (immature specimen) from the state of Puebla, with *Loxoscelesmalintzi* sp. n. being the third species from the state (Figs 75, 78). However, in the case of *L.zapoteca*, males from Puebla are unknown, so we cannot corroborate the accurate identity of the species. In the collected material of *L.malintzi* sp. n. from localities near Acatlán de Osorio where *L.zapoteca* was reported (Fig. 75), only males of the new species were collected but no males of *L.zapoteca*. Also, although there is high variation in the seminal receptacles in *L.malintzi* (Figs 63–67), the seminal receptacles are completely different from those of *L.zapoteca* (Gertsch and Ennik 1983: figs 48–52). Also, the male palp and female genitalia are different in both species (Gertsch and Ennik 1983: figs 32–35, 48–51). The record of *L.boneti* from Puebla is also doubtful: the specimen is an immature, and the type locality of *L.boneti* is Acapulco, Guerrero, 250 km from Puebla (Fig. 75).

###### Natural history.

The specimens of *Loxoscelesmalintzi* sp. n. were collected in a tropical deciduous forest (Figs 11–16). The micro habitat where the specimens were collected was under and among large rocks, and inside of rotten and dry cactus of the genus *Opuntia* and *Pachycereus* (arrows, Figs 13, 14, 17). At the type locality, the specimens were collected close together on a live large cactus (*Pachycereus*). They were collected at night when males are more active. These specimens were collected at 1.5–2.0 m high in the live cactus where their webs where located. In addition, the new species has anthropogenic habits: the specimens from San Pablo Anicano, Puebla were collected inside a house, under a concrete laundry sink and among concrete blocks in a yard. Even an adult male was collected at night walking on the kitchen floor of the house.

###### Distribution.

MEXICO: Puebla, Morelos, Guerrero (Figs 75, 78).

**Figures 1–5.**
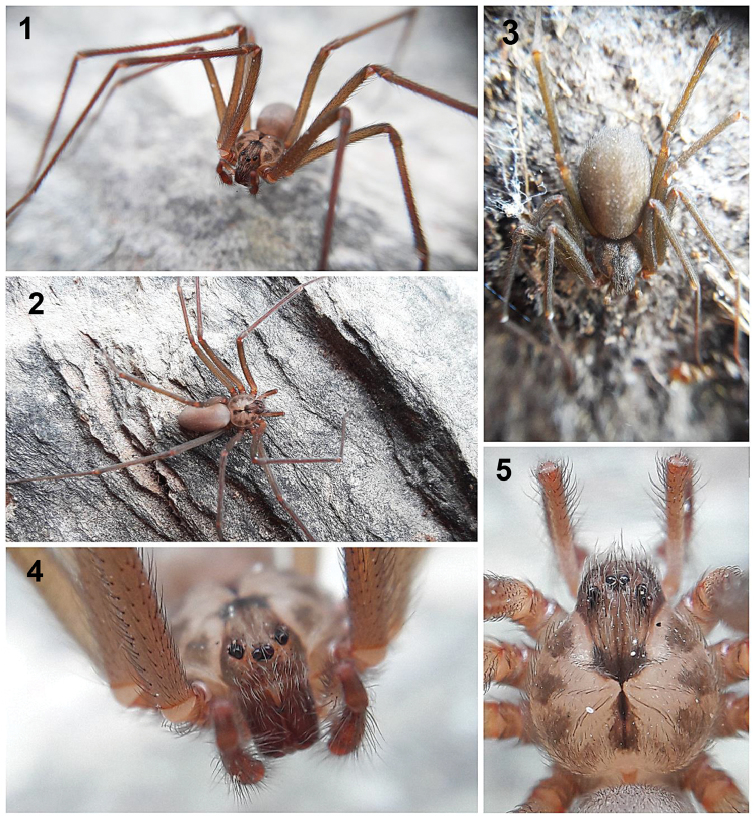
Live specimens of *Loxoscelesmalintzi* sp. n. from the type locality: 1.5 km NE of Panteón de San Pablo Anicano, Municipality San Pablo Anicano, Puebla, Mexico**1, 2, 4, 5** Male holotype (CNAN-T01262) **3** Female paratype (CNAN-T01264). Photos by Alejandro Valdez-Mondragón (2018).

**Figures 6–10.**
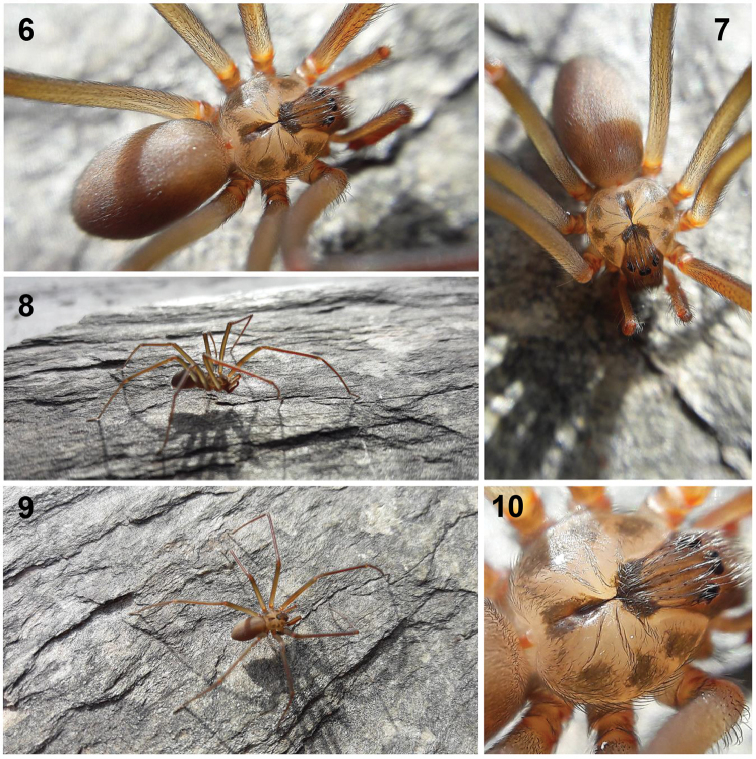
Live male of *Loxoscelesmalintzi* sp. n. from 1.5 km SE of Guadalupe Alchipin, Mpio, Ahuehuetitla, Puebla, Mexico. Photos by Alejandro Valdez-Mondragón (2018).

**Figures 11–17.**
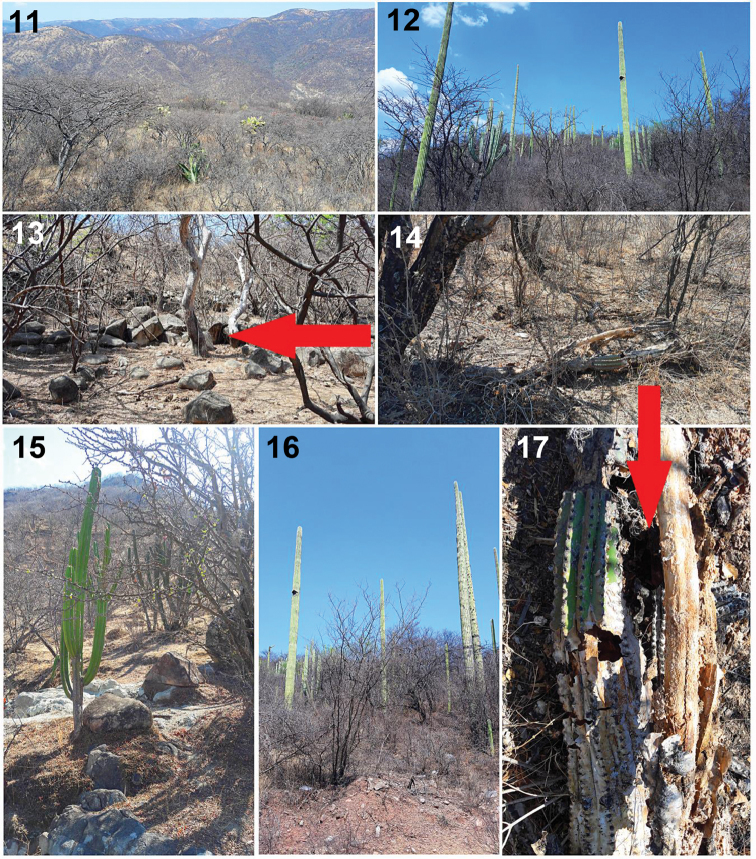
Habitat and microhabitat of *Loxoscelesmalintzi* sp. n. **11–14, 17** Tropical deciduous forest from of the type locality: 1.5 km NE of Panteón de San Pablo Anicano, Municipality San Pablo Anicano, Puebla, Mexico (arrows indicate the micro habitat where the specimens can be found, under big rocks and inside of a rotten and dry cactus in the ground of the genus *Pachycereus*) **15, 16** Tropical deciduous forest from 1.5 km SE of Guadalupe Alpichin, Municipality Ahuehuetitla, Puebla, Mexico. Photos by Alejandro Valdez-Mondragón (2017).

**Figures 18–21.**
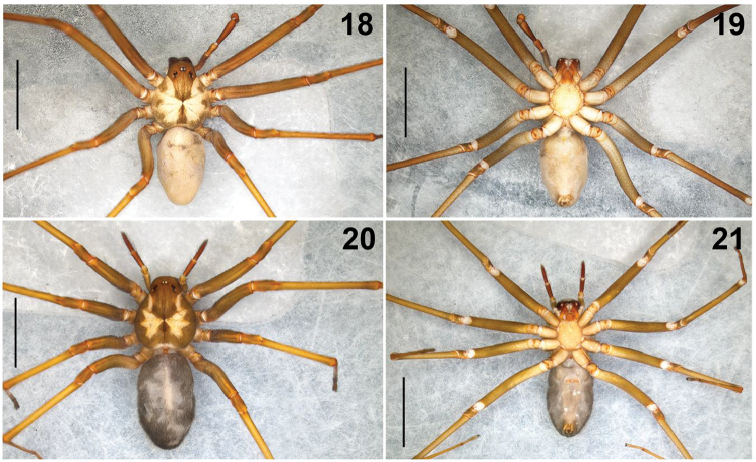
*Loxoscelesmalintzi* sp. n. **18, 19** Habitus of male holotype, dorsal and ventral views, respectively **20, 21** Habitus of female paratype, dorsal and ventral views, respectively. Scale bar: 1 mm.

**Figures 22–27.**
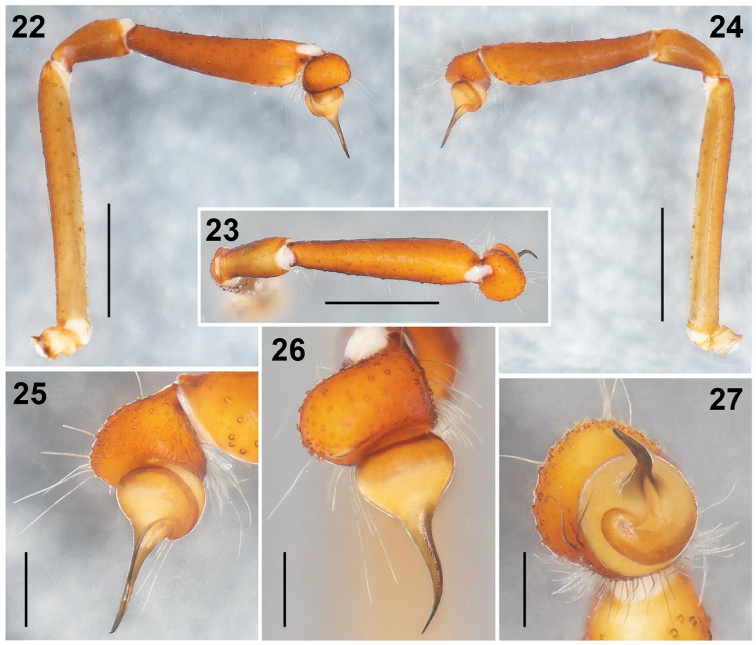
*Loxoscelesmalintzi* sp. n. Male Holotype: **22–24** Left palp, prolateral, dorsal and retrolateral views respectively **25–27** Detail of the bulb and embolus, retrolateral, dorsal and apical views, respectively. Scale bars: 1 mm (**22–24**), 0.5 mm (**25–27**).

**Figures 28–31.**
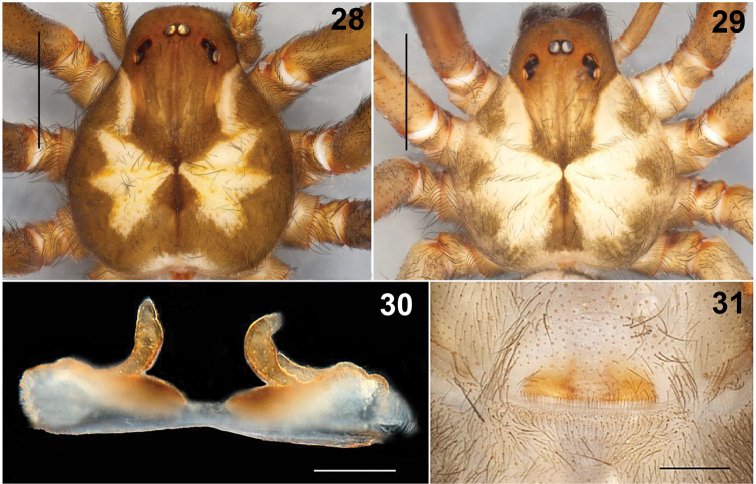
*Loxoscelesmalintzi* sp. n. **28, 29** Caparace of female paratype and male holotype, respectively. Female Paratype: **30** Seminal receptacles **31** Genital area, ventral view. Scale bars: 1 mm (**28, 29, 31**), 0.2 mm (**30**).

**Figures 32–37.**
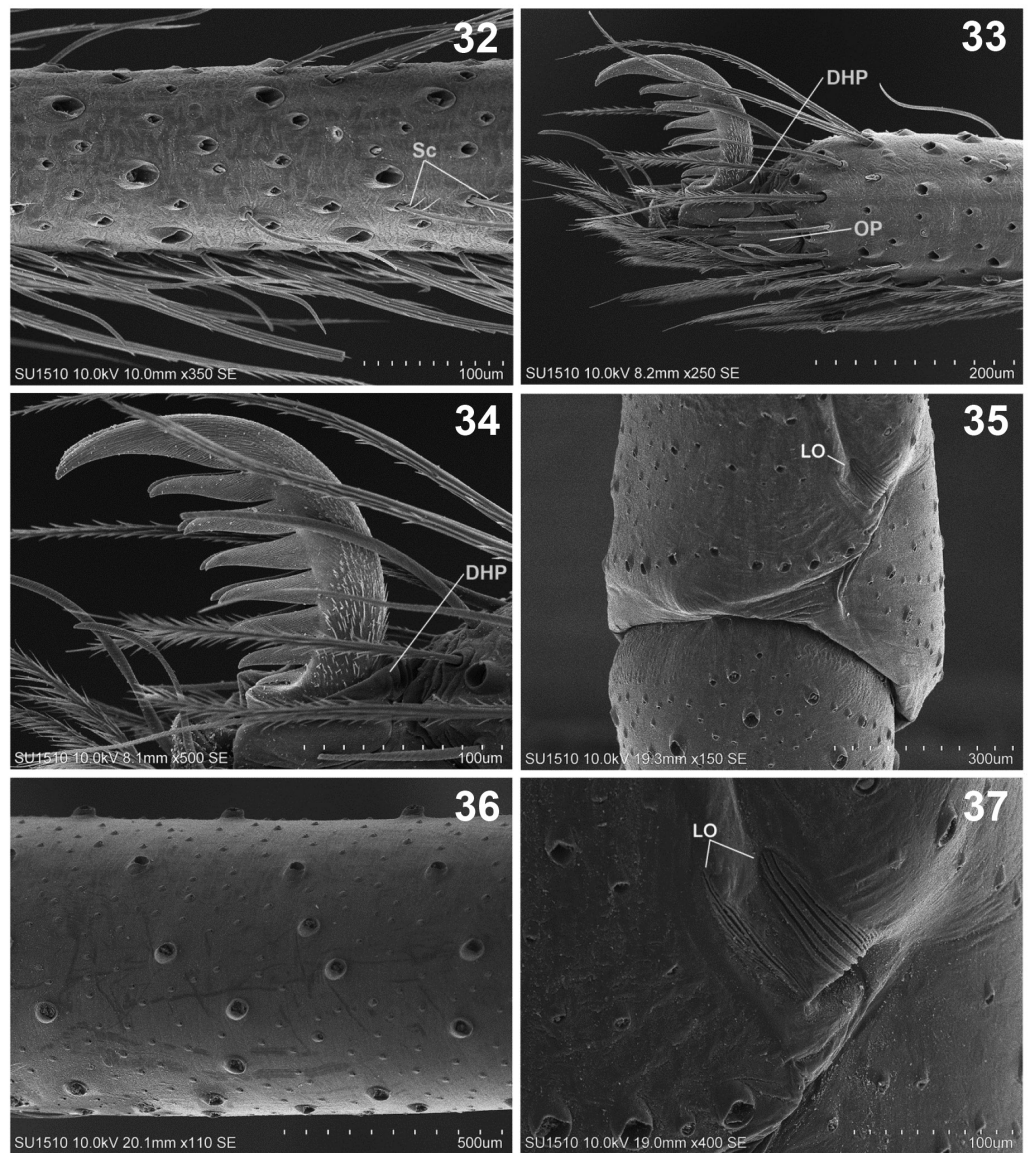
*Loxoscelesmalintzi* sp. n. Male **32** Right metatarsus I, retrolateral view, showing different type of setae insertions **33** Right tarsus I, prolateral view, showing the claws **34** Detail of claws and setae **35** Right leg I, ventral view of patella and tibia, showing the lyriform organ (LO) **36** Right femur I, retrolateral view **37** Detail of LO of patella I. Abbreviations: DHP, dorsal hood of podotarsite; LO, lyriform organ; OP, open podotarsite; Sc, scale (seta).

**Figures 38–43.**
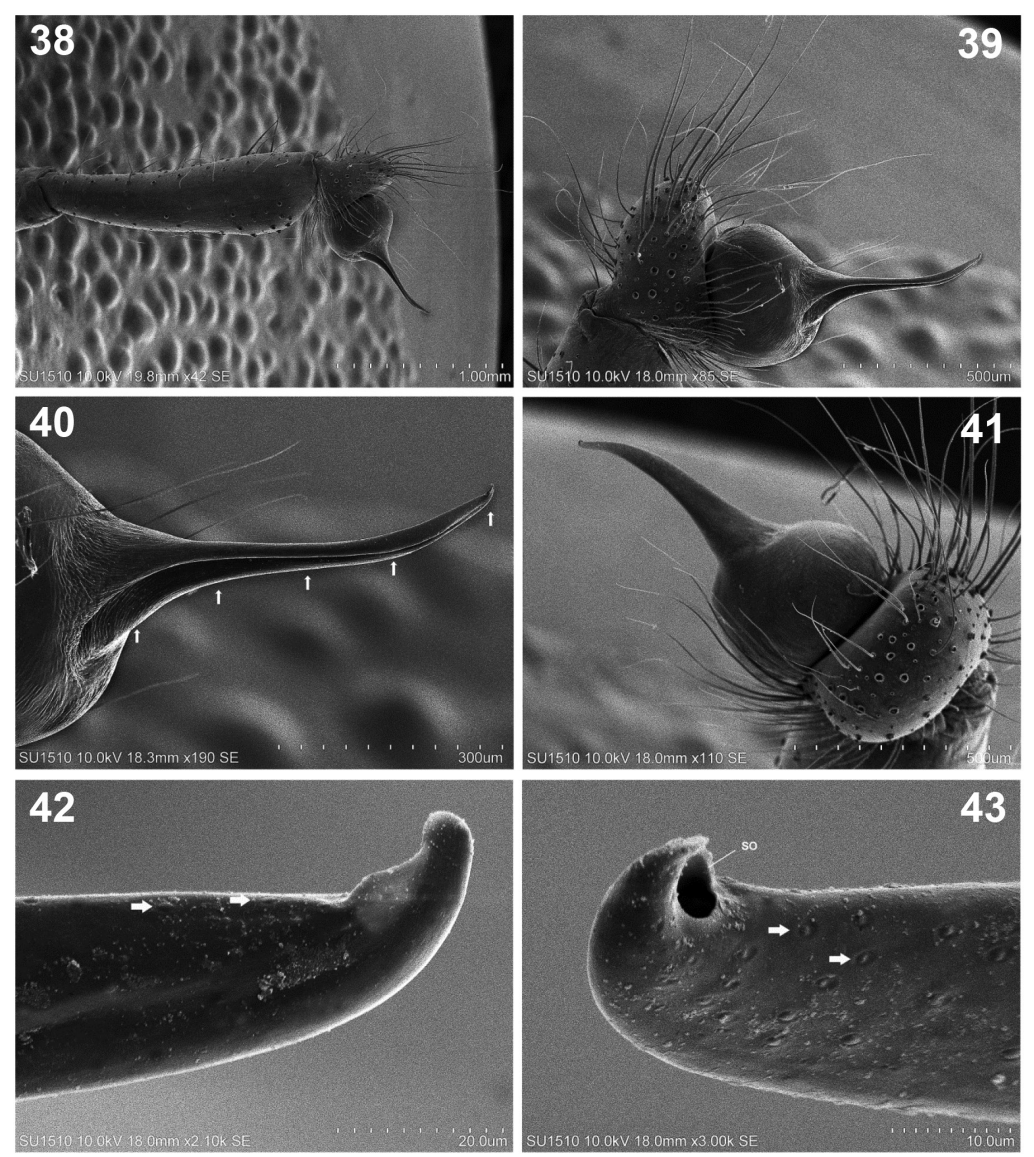
*Loxoscelesmalintzi* sp. n. Male **38** Left palp, prolateral view **39** Detail of tarsus, bulb and embolus **40** Detail of the embolus (arrows indicate the canal along the embolus) **41** Detail of tarsus, bulb and embolus, dorsal view **42, 43** Embolus tip, prolateral and retrolateral views respectively, showing the spermatic outlet (arrows indicate cuticular marks, unknown function). Abbreviations: SO, spermatic outlet

**Figures 44–49.**
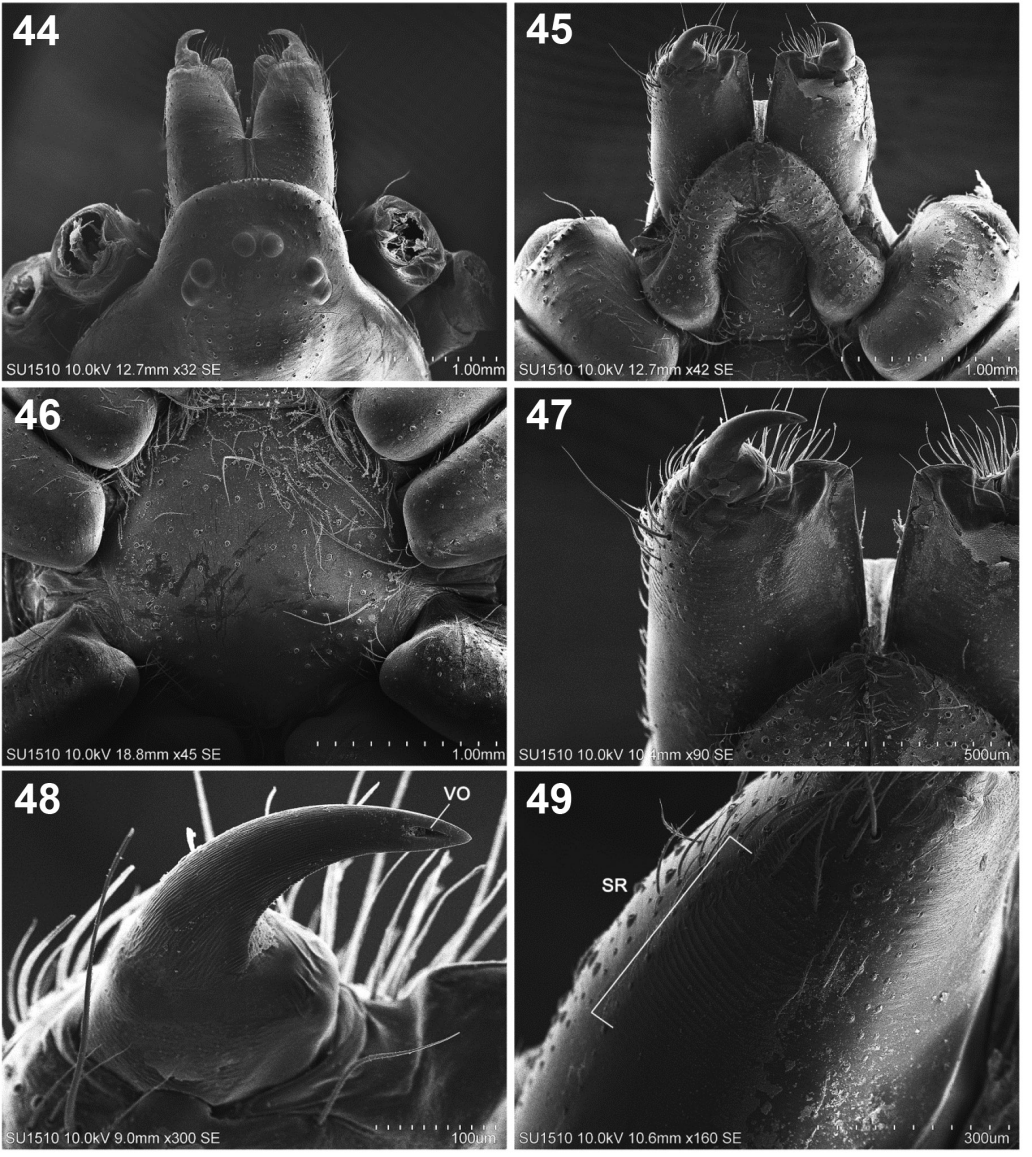
*Loxoscelesmalintzi* sp. n. Male **44** Anterior part of carapace and chelicerae, dorsal view **45** Chelicerae, endites and labium, ventral view **46** Detail of sternum **47** Detail of right chelicera, posterior view **48** Detail of right fang of chelicerae, showing the venom outlet **49** Detail of stridulatory ridges of right chelicerae. Abbreviations: SR, stridulatory ridges; VO, venom outlet.

**Figures 50–54.**
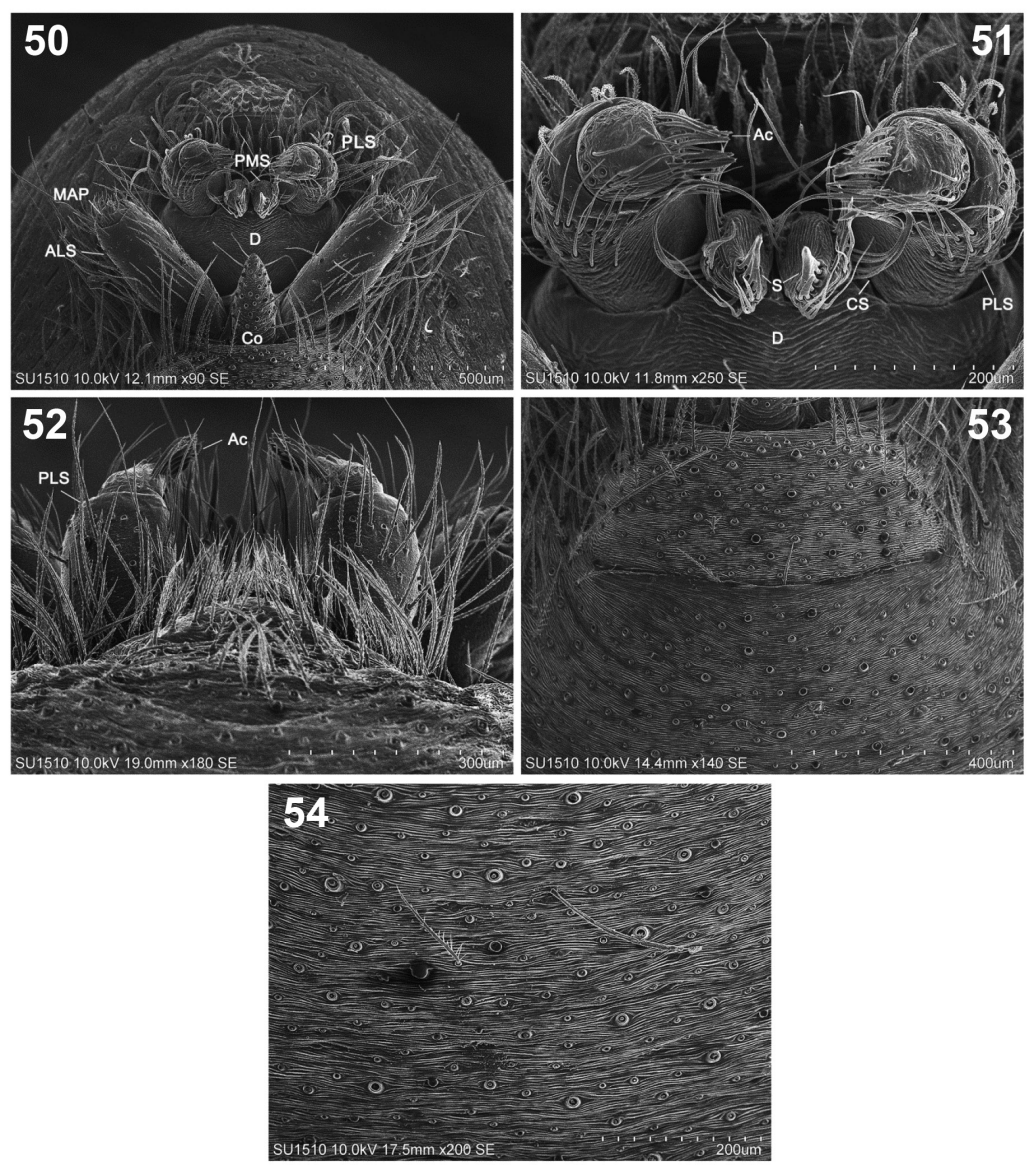
*Loxoscelesmalintzi* sp. n. Male **50** Spinnerets **51** Detail of PMS and PLS **52** PLS, anterior view **53** Detail of the tracheae **54** Detail of the cuticle of the opisthosoma. Abbreviations: Ac, aciniform gland spigot; ALS, anterior lateral spinnerets; CS, curved spigot; Co, colulus; D, diastema; MAP, major ampullate glands; PLS, posterior lateral spinnerets; PMS, posterior median spinnerets; S, spigot.

**Figures 55–62.**
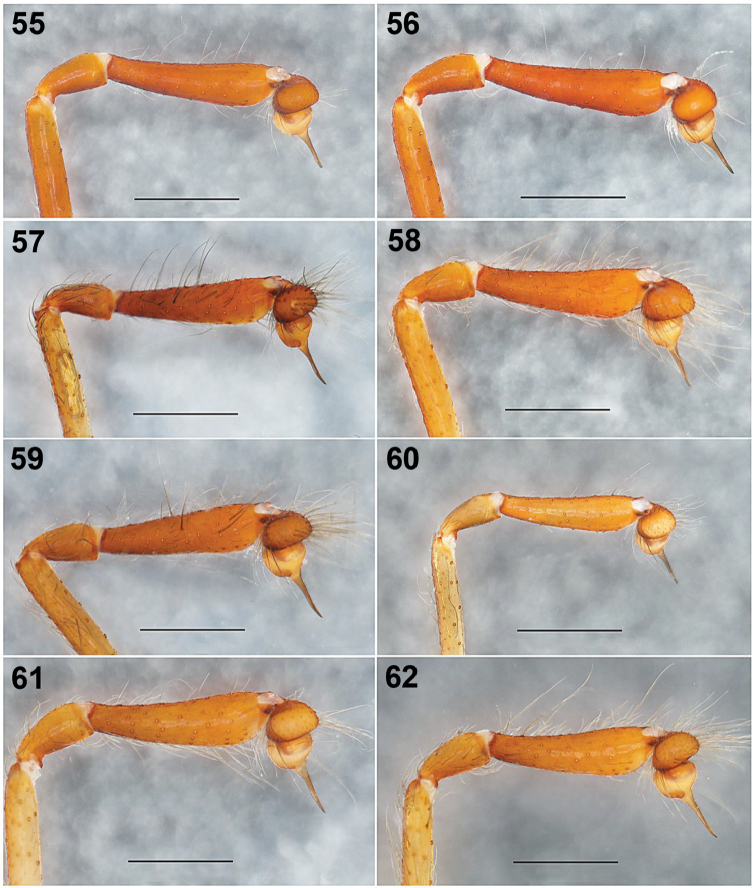
*Loxoscelesmalintzi* sp. n. Variation of the male palps, left palps, prolateral views **55, 56** 1.5 km al NE del Panteón de San Pablo Anicano, Municipality San Pablo Anicano, Puebla (type locality) **57** 2km al S de Agua Escondida, Municipality Izúcar de Matamoros, Puebla **58, 59** Biological Station El Limón Cuachichinola, Municipality Tepalcingo, Morelos **60** Road to Mexcaltepec viejo, Municipality Taxco de Alarcón, Guerrero **61** Ticumán, Municipality Tlaltizapán, Morelos **62** 2 km al Oeste de Ahuelican “Cerro de la Coronilla”, Municipality Tepecuacuilco de Trujano, Guerrero. Scale bar: 0.5 mm.

**Figures 63–67.**
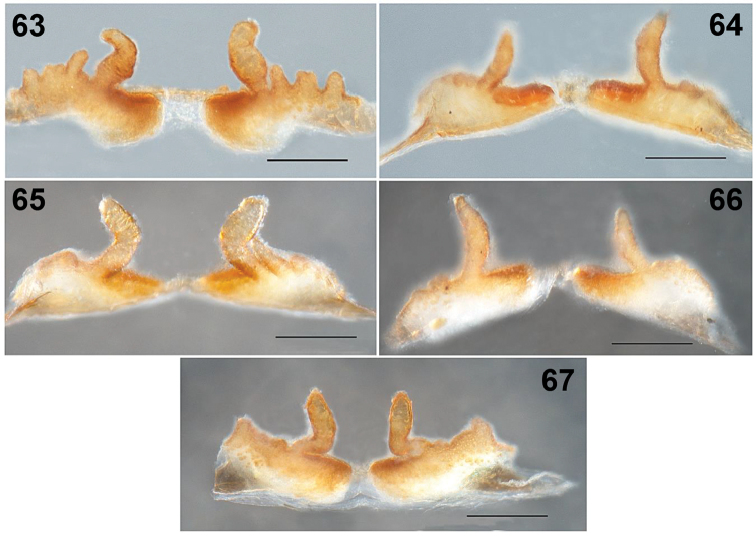
*Loxoscelesmalintzi* sp. n. Variation of the seminal receptacles of the females, dorsal views. Puebla **63, 64** Insurgentes Street, cerrada Insurgentes, Section San Juan, San Pablo Anicano, Municipality San Pablo Anicano **65, 66** 1.5 km al NE del Panteón de San Pablo Anicano, Municipality San Pablo Anicano. Guerrero **67** Road to Mexcaltepec viejo, Municipality Taxco de Alarcón. Scale bar: 0.2 mm.

##### Loxosceles rufescens

Taxon classificationAnimaliaAraneaeSicariidae

(Dufour, 1820)

Figs 68–72

Scytodes rufescens Dufour 1820c: 203, pl. 76, fig. 5 (male).
Loxosceles citigrada Heineken and Lowe in Lowe (transferred) 1832: 322, pl. 48, figs 1–14 (male, female).
See World Spider Catalog (2018) for complete records.

###### Material examined.

MEXICO: *Chihuahua*: 1 male, 1 female (LATLAX-Ara0183) from Instituto de Biomédicas de la Universidad Autónoma de Ciudad Juárez (31. 74645, −106.4444; 1130 m), Municipality Ciudad Juárez, no date, P. Flores col.

###### Diagnosis.

**Figures 68–72.**
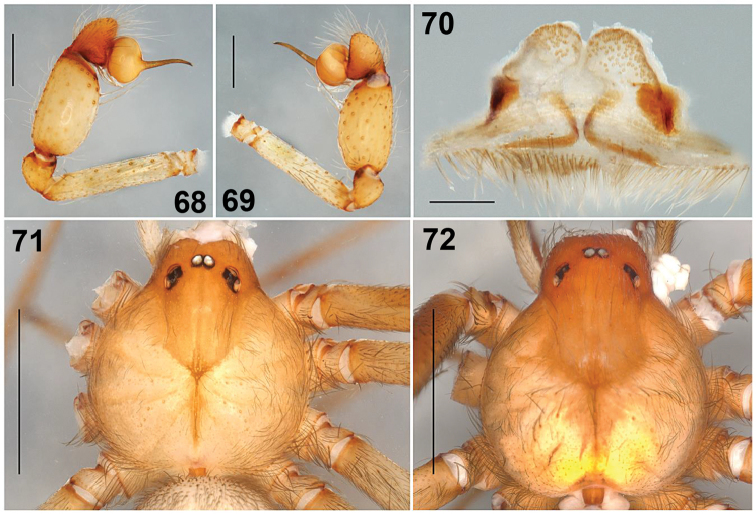
*Loxoscelesrufescens* Dufour, 1820, from Ciudad Juárez, Chihuahua, Mexico**68, 69** Male, left palp, prolateral and retrolateral views respectively **70** Female, seminal receptacles, dorsal view **71, 72** Caparace dorsal view, male and female views respectively. Scale bars: 0.5 mm (**68, 69**), 0.2 mm (**70**), 1 mm (**71, 72**).

*L.rufescens* resembles *Loxoscelesfoutadjalloni* Millot, 1941; in having male palpal tibia wide and a long embolus (Lotz 2012: fig. 9C), however in *L.rufescens* the male palp tibia is wider and the embolus is sigmoid-shaped (Figs 68–69), whereas in *L.foutadjalloni* the embolus is long and curved (Lotz 2012: fig. 9C). Females resemble *L.foutadjalloni* by the shape of the seminal receptacles (Lotz 2012: fig. 10B), however in *L.rufescens* they are short and round distally (Fig. 70), whereas in *L.foutadjalloni* the seminal receptacles are longer and distally bifurcated and rounded (Lotz 2012: fig. 10B).

###### Description.

See Chomphuphuang et al. (2016).

###### Distribution.

*Loxoscelesrufescens* (Figs 68–72) has a natural distribution in the Mediterranean Basin and the Middle East (Nentwig et al. 2017; Tahami et al. 2017), but also is considered a cosmopolitan species (Nentwig et al. 2017; World Spider Catalog 2018).

###### Remarks.

**Figures 73–74.**
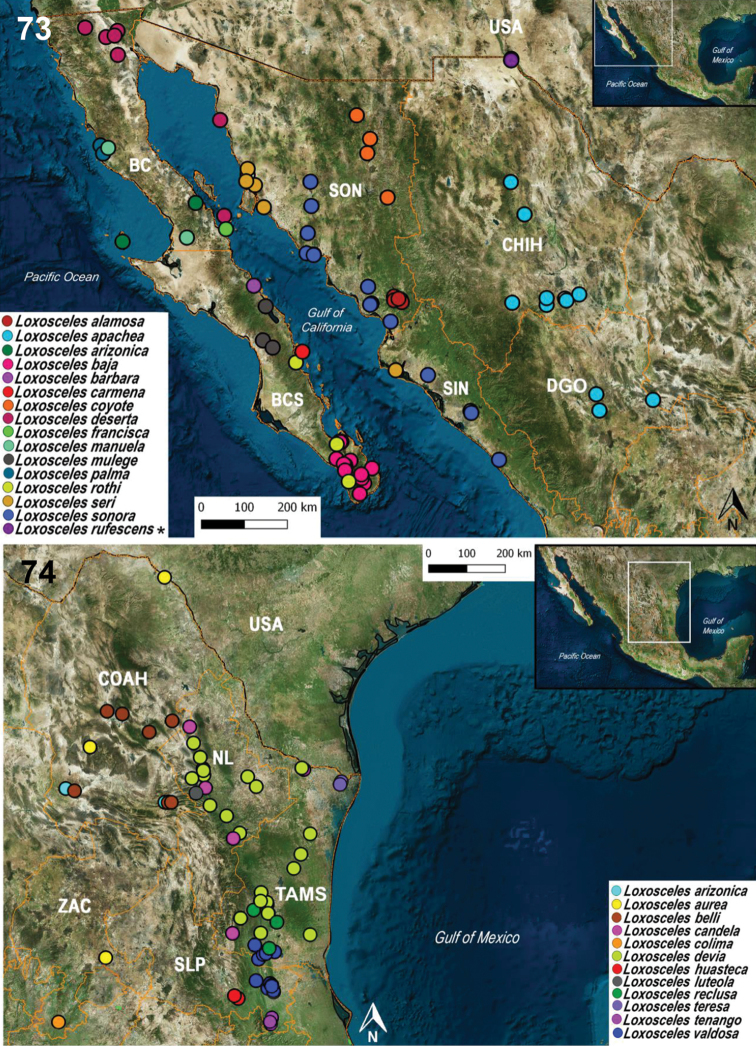
Updated records of the species of *Loxosceles* for the states of north of Mexico. Asterisk in *L.rufescens* represents a new record for Mexico. Abbreviations for the Mexican states: BC, Baja California; BCS, Baja California Sur; CHIH, Chihuahua; COAH, Coahuila; DGO, Durango; NL, Nuevo León; SIN, Sinaloa; SLP, San Luís Potosí; SON, Sonora; TAMS, Tamaulipas; ZAC, Zacatecas.

In Mexico, *L.rufescens* is only known from two records, from the states of Tamaulipas and Chihuahua (Fig. 73). Chickering (1937) reported *L.rufescens* from San Carlos Mountains, Tamaulipas; however, he never described or illustrated any specimen, which makes his record questionable (Fig. 74).

###### Updated distribution records for the 39 species of Loxosceles from Mexico.

**Figures 75–76.**
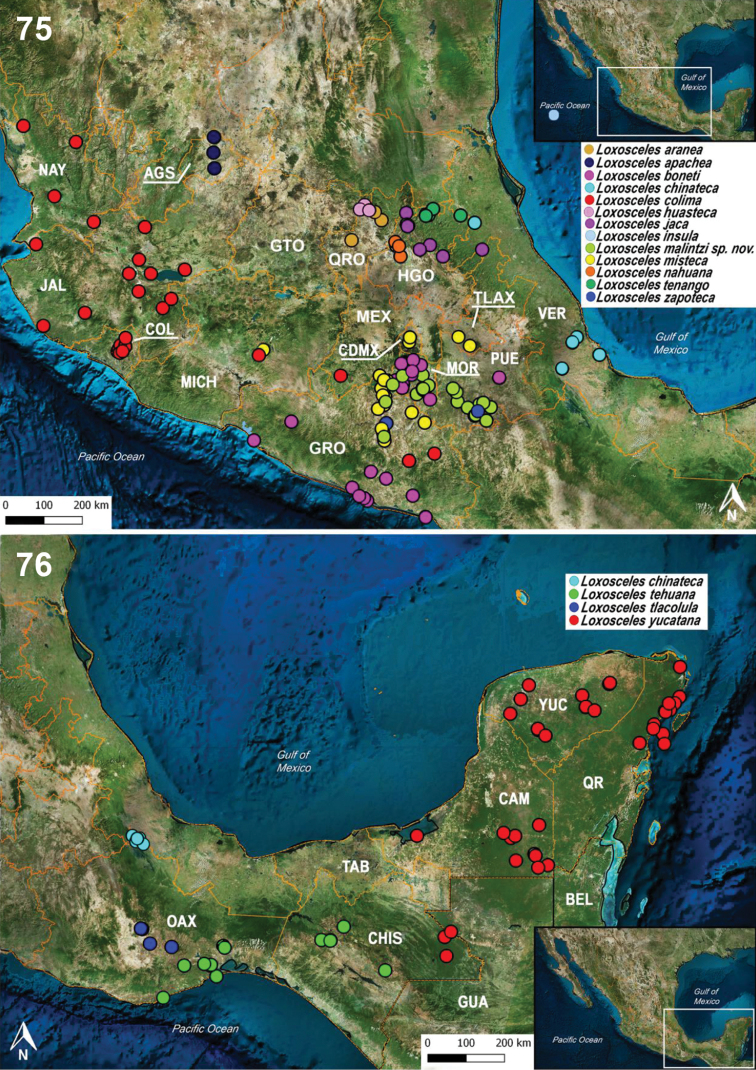
Updated records of the species of *Loxosceles* for the states of central region and south of Mexico. Abbreviations for the Mexican states: AGS, Aguascalientes; CAM, Campeche; CDMX, Mexico City; CHIS, Chiapas; COL, Colima; MEX, Estado de México; GTO, Guanajuato; GRO, Guerrero; HGO, Hidalgo; JAL, Jalisco; MICH, Michoacán; MOR, Morelos; NAY, Nayarit; OAX, Oaxaca; PUE, Puebla; QR, Quintana Roo; QRO, Querétaro; TAB; Tabasco; TLAX, Tlaxcala; VER, Veracruz; YUC, Yucatán.

A total of 461 records of the 39 species of *Loxosceles* distributed in Mexico were reviewed. Twenty records were discarded for not having complete localities or having doubtful georeferences. Thus, a total of 441 records were used to make the distribution maps (Figs 73–76). The states with the most records are Guerrero with 55, Morelos with 35, and Baja California Sur with 30 (Fig. 75). The state of Tabasco only has a single record (Appendix 1, Fig. 75). The most diverse states are Baja California Sur, Baja California, Sonora (with five species each), Guerrero, Tamaulipas (with four species each), and Oaxaca, Puebla, Hidalgo, Coahuila, San Luis Potosí, Nuevo León (with three species each) (Figs 73, 74). The least diverse states are Durango, Zacatecas, Michoacán, Querétaro, Chihuahua and Sinaloa (with two species each); Jalisco, Guanajuato, Quintana Roo, Colima, Chiapas, Yucatan, Campeche, Tabasco, Veracruz, Mexico City, Nayarit, Aguascalientes, Tlaxcala and state of Mexico (with a single species each) (Figs 73–76).

Regarding the number of total records per species of *Loxosceles*, the species with the most records are *L.boneti* with 59 and *L.colima* with 57 (Fig. 75). The species with the least number of records are *L.barbara, L.carmena, L.francisca, L.insula, L.luteola* and *L.rufescens* with a single record each (Fig. 74). A new record of *L.misteca* was found for Tlaxcala (Figs 75). A third record of *L.reclusa* was found for Tamaulipas (Fig. 74). The record of *L.rufescens* from Ciudad Juárez, Chihuahua represents the second record for the country of this introduced species and the first well-documented and illustrated record from Mexico (Figs 68–72, 73, Appendix 1).

**Figures 77–78.**
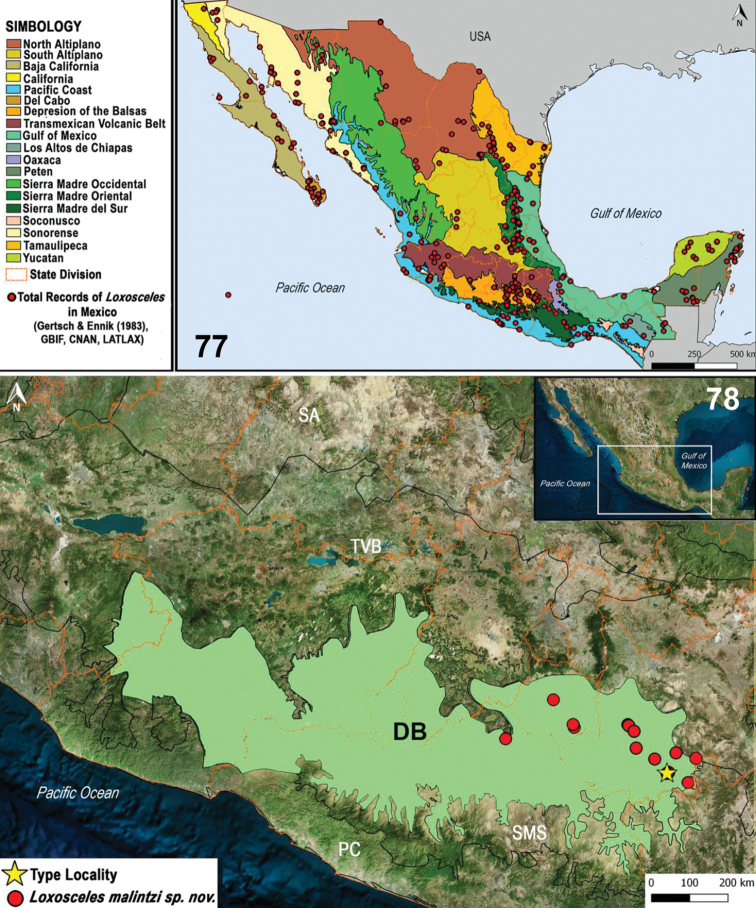
**77** Biogeographical provinces of Mexico showing the distribution records of the genus *Loxosceles***78** Known records of *Loxoscelesmalintzi* sp. n. from the Depression of the Balsas province (green area), including the type locality.

## Discussion

All 32 states of the Mexican Republic, including Mexico City, have records of some native or introduced species of *Loxosceles* (Figs 73–77). Regarding the distribution of species of *Loxosceles* in Mexico, although the highest diversity of species is in the northwest, more field work is necessary to collect additional material, mainly from the Baja California Peninsula where the species have been described based on one specimen of one sex (male or female) or few specimens (Fig. 73) (Gertsch and Ennik 1983).

Regarding the introduced species in Mexico, the record of *L.rufescens* from Ciudad Juárez, Chihuahua (Fig. 73), a widely distributed species throughout the Mediterranean Basin and the Middle East (Nentwig et al. 2017; Tahami et al. 2017), is the first well documented and illustrated record of this species from Mexico (Figs 68–72). The third record of *L.reclusa* from Mexico was found for the state of Tamaulipas. The first two records were recorded by Gertsch and Ennik (1983) (Fig. 74). *Loxoscelesreclusa* is an introduced species. The natural distribution is from the south-central United States, from southern Illinois south to Texas and from eastern Tennessee west to Kansas (Saupe et al. 2011: fig. 2A, B). The records of *L.arizonica* from Coahuila state are doubtful (Fig. 74) because the natural distribution of the species is from Arizona, USA. For the central region of Mexico, some of the records are introduced species in temperate climates and mainly in urban zones. Such is the case of *L.misteca* in Mexico City and Tlaxcala (reported for the first time) (Fig. 75). The type locality of *L.misteca* is from Taxco de Alarcón, in Guerrero state; it is a common species in tourist caves of the state such as Grutas de Cacahuamilpa, Grutas del Mogote, Pozo Melendez, and Cave of Carlos Pacheco. These caves are located in a tropical deciduous forest, a habitat preferred by many species of *Loxosceles* from Mexico, mainly from the Pacific region (Figs 11–17). Regarding the species of *Loxosceles* from Mexico City, Durán-Barrón and Ayala-Islas (2007) and Durán-Barrón et al. (2009) reported two species: *L.misteca* and one undetermined species (*Loxosceles* sp.), probably a immature specimen of *L.misteca.* Also, Gertsch (1958) recorded *L.nahuana* for Mexico City based on an adult female; however, Gertsch and Ennik (1983) only cited specimens of *L.nahuana* from Hidalgo state where this species is distributed (Fig. 75), which makes the record this species in Mexico City questionable.

According to the biogeographical scheme for Mexico by Morrone (2004, 2005), all biogeographical provinces have recorded species of *Loxosceles* (Fig. 77). The highest diversity of species of *Loxosceles* from Mexico is towards the north, and the diversity tends to decrease towards the south of the country (Figs 73–76). The records of *Loxosceles* from Mexico are located mainly in biogeographical provinces of lowlands and in dry and tropical forests, including tropical deciduous forests, and also deserts, such as Baja California, Del Cabo, Sonorense, North Altiplano, Pacific Coast, Sierra Madre del Sur and Depression of the Balsas provinces (where *L.malintzi* sp. n. is distributed, Fig. 78) (Fig. 77). Although most of the species of *Loxosceles* from Mexico are distributed in tropical deciduous forest (Figs 11–17), species such as *L.chinateca* and *L.yucatana* are distributed in tropical rain forests. *Loxosceleschinateca* is from the states of Oaxaca and Veracruz (Gulf of Mexico and Oaxaca provinces), whereas *L.yucatana* is from the states of Chiapas, Tabasco and Yucatan Peninsula (Gulf of Mexico, Peten and Yucatan provinces) (Fig. 76). The records of *Loxosceles* in biogeographical provinces with mountains at high elevations (> 2000 m.a.s.l.), temperate climates, and with pine, oak or oak-pine forest are scarce. Such is the case of the Sierra Madre Occidental, high-lands of North Altiplano, South Altiplano, Transmexican Volcanic Belt and Los Altos de Chiapas provinces, where some records of *Loxosceles* might be those of introduced species (Fig. 77). This idea is supported by ecological niche modeling for the species of *Loxosceles* from Mexico (in press). In the case of the Sierra Madre Oriental province, composed of high mountains and temperate and mountain mesophyll forests, the records of *Loxosceles* are mainly from the east of the province where the elevations are lower and the climate is more tropical (Fig. 77). Many of these records are from karstic caves, one of the preferred microhabitats of some species from Mexico (e.g. *L.misteca, L.boneti, L.chinateca, L.tehuana, L.tenango* and *L.yucatana*).

## Supplementary Material

XML Treatment for Loxosceles malintzi

XML Treatment for Loxosceles rufescens

## Appendix 1

**Table:** List of the Mexican species of *Loxosceles* and records per state. *New records for Mexico. ** New records for State.

Species	Author(s), year	Records	States
* L. alamosa *	Gertsch & Ennik, 1983	4	Son
* L. apachea *	Gertsch & Ennik, 1983	20	Ags, Chih, Dgo
* L. aranea *	Gertsch, 1973	4	Qro
* L. arizonica *	Gertsch & Mulaik, 1940	18	BC, Coah
* L. aurea *	Gertsch, 1973	5	Coah, Zac
* L. baja *	Gertsch & Ennik, 1983	13	BCS
* L. barbara *	Gertsch & Ennik, 1983	1	BCS
* L. belli *	Gertsch, 1973	7	Coah
* L. boneti *	Gertsch, 1958	59	Gro, Mor, Pue
* L. candela *	Gertsch & Ennik, 1983	3	NL, Tams
* L. carmena *	Gertsch & Ennik, 1983	1	BCS
* L. chinateca *	Gertsch & Ennik, 1983	12	Oax, Ver
*L.colima***	Gertsch, 1958	57	Col, Gro, Jal, Mex, Mich**, Nay, Zac
* L. coyote *	Gertsch & Ennik, 1983	3	Son
* L. deserta *	Gertsch, 1973	10	BC, Son
* L. devia *	Gertsch & Mulaik, 1940	31	NL, Tams
* L. francisca *	Gertsch & Ennik, 1983	1	BC
* L. huasteca *	Gertsch & Ennik, 1983	5	Gto, Qro, SLP
* L. insula *	Gertsch & Ennik, 1983	1	Col
* L. jaca *	Gertsch & Ennik, 1983	8	Hgo
* L. luteola *	Gertsch, 1973	1	NL
*L.malintzi* sp. n.		25	Gro, Mor, Pue
* L. manuela *	Gertsch & Ennik, 1983	2	BC
*L.misteca***	Gertsch, 1958	18	CDMX, Gro, Mich**, Mor, Tlax**
* L. mulege *	Gertsch & Ennik, 1983	3	BCS
* L. nahuana *	Gertsch, 1958	7	Hgo
* L. palma *	Gertsch & Ennik, 1983	2	BC
* L. reclusa *	Gertsch & Mulaik, 1940	3	Tams
* L. rothi *	Gertsch & Ennik, 1983	3	BCS
*L.rufescens**	Dufour, 1820	1	Chih*
* L. seri *	Gertsch & Ennik, 1983	6	Sin, Son
* L. sonora *	Gertsch & Ennik, 1983	13	Sin, Son
* L. tehuana *	Gertsch, 1958	17	Chis, Oax
* L. tenango *	Gertsch, 1973	9	Hgo, SLP
* L. teresa *	Gertsch & Ennik, 1983	3	Tams
* L. tlacolula *	Gertsch & Ennik, 1983	4	Oax
* L. valdosa *	Gertsch, 1973	11	SLP, Tams
* L. yucatana *	Chamberlin & Ivie, 1938	41	Chis, Tab, Cam, Yuc, QR
* L. zapoteca *	Gertsch, 1958	9	Pue, Gro
**TOTAL**		**441**	

Ags: Aguascalientes, BC: Baja California, BCS: Baja California Sur, Cam: Campeche, CDMX: Mexico City, Coah: Coahuila, Col: Colima, Chis: Chiapas, Chih: Chihuahua, Dgo: Durango, Gto: Guanajuato, Gro: Guerrero, Hgo: Hidalgo, Jal: Jalisco, Mexico: State of Mexico, Mich: Michoacan, Mor: Morelos, Nay: Nayarit, NL: Nuevo Leon, Oax: Oaxaca, Pue: Puebla, Qro: Queretaro, QR: Quintana Roo, SLP: San Luis Potosi, Sin: Sinaloa, Son: Sonora, Tab: Tabasco, Tams: Tamaulipas, Tlax: Tlaxcala, Ver: Veracruz, Yuc: Yucatan, Zac: Zacatecas.
